# Prevalence of human papillomaviruses in self-collected samples among women attending antenatal care in Ethiopia: a cross-sectional study

**DOI:** 10.3332/ecancer.2024.1739

**Published:** 2024-08-14

**Authors:** Isabel Runge, Johanna M A Klein, Ann-Katrin Pannen, Semaw Abera, Tariku Wakuma, Yirgu Gebrehiwot, Susanne Unverzagt, Andreas Wienke, Christoph Thomssen, Andreas M Kaufmann, Ahmedin Jemal, Tamrat Abebe, Dana Holzinger, Tim Waterboer, Daniela Höfler, Adamu Addissie, Eva Johanna Kantelhardt

**Affiliations:** 1 Department of Gynaecology, Martin-Luther-University Halle-Wittenberg, 06108 Halle, Germany; 2 Global Health Working Group, Institute of Medical Epidemiology, Biometrics and Informatics, Martin-Luther-University Halle-Wittenberg, 06108 Halle, Germany; 3 Department of Radiation Oncology, Faculty of Medicine, Martin-Luther-University Halle-Wittenberg, 06108 Halle (Saale), Germany; 4 School of Public Health, College of Health Sciences, Mekelle University, Mekele 0231, Ethiopia; 5 Kilte Awlaelo – Health and Demographic Surveillance Site, College of Health Sciences, Mekelle University, Mekele 0231, Ethiopia; 6 Aira Hospital, Aira, Ethiopia; 7 Department of Obstetrics and Gynaecology, College of Health Sciences, Addis Ababa University, Addis Ababa 1000, Ethiopia; 8 Institute of General Practice and Family Medicine, Center of Health Sciences, Martin-Luther-University Halle-Wittenberg, 06097 Halle (Saale), Germany; 9 Clinic for Gynecology, Charité – Universitätsmedizin Berlin, Corporate Member of Freie Universität Berlin, Berlin Institute of Health, Campus Virchow Klinikum, Humboldt-Universität zu Berlin, 10117 Berlin, Germany; 10 American Cancer Society, Atlanta, GA 30303, USA; 11 Department of Microbiology, Immunology & Parasitology, School of Medicine, College of Health Sciences, Addis Ababa University, Addis Ababa 1000, Ethiopia; 12 Infections and Cancer Epidemiology, German Cancer Research Center (DKFZ), 69120 Heidelberg, Germany; 13 School of Public Health, College of Health Sciences, Addis Ababa University, Addis Ababa 1000, Ethiopia

**Keywords:** cervical cancer screening, HPV test, Ethiopia, pregnancy, population-based, cervical cancer prevention, Africa

## Abstract

Cervical cancer is the second most commonly diagnosed cancer in women in Ethiopia. However, data are limited on the prevalence of human papillomavirus (HPV) genotypes. Self-sampled vaginal lavages were obtained consecutively from 783 women attending 7 health facilities across Ethiopia. Genotype prevalence was assessed by Multiplex-Papillomavirus-Genotyping which detects and individually identifies 51 genotypes and 3 subtypes. Genotype-specific prevalence was described and associations with known risk factors were analysed. The overall HPV prevalence (age range 18–45) was 33.1% (95% confidence interval (CI) 29.8–36.4). The prevalence of HPV was different in the rural and urban population with 17.6% (95%CI 11.6–23.7) and 36.8% (95%CI 33.1–40.6) (p < 0.001 chi-square test), respectively. The most common high-risk types were HPV 16 (6.6%), followed by HPV 52 (4.3%), 51 and 39 (both 2.9%). Urban women compared to rural women had a higher risk of being HPV positive (odds ratio 2.36 (95% CI 1.47–3.79; p < 0.001). Age at sexual debut ≤15 years and polygamous husband (in urban women) also increased the risk of being HPV positive nearly two-fold. The high prevalence of hr-HPV in Ethiopian women in the reproductive age group shows the need for screening programs. The nonavalent HPV vaccine covers the most prevalent hr-HPV genotypes as found in this study and can therefore be used effectively. Since antenatal care is the best-utilised health service, implementing self-sampled vaginal lavage could be an opportunity for screening in this age group. Screening algorithms and triage still need to be defined to avoid over-treatment in these women.

## Background

### Importance of cervical cancer

Cervical cancer was responsible for an estimated 311,365 deaths worldwide in 2018 [4]; of those, the majority occurred in less developed regions. It is one of the diseases with high disparity in survival between the different countries. Cancer death rates are higher for women in low- and middle-income countries. East Africa has one of the highest age-standardised mortality rates with 30 per 100,000 women [4, 34]. A reason is the lack of adequate screening facilities and therefore low screening uptake, correct diagnosis and treatment; especially in sub-Saharan Africa despite the known effectiveness of prevention by screening programs [40].

### Risk factors and preventive measures

The main risk factor for developing cervical cancer is persistent infection with high-risk (hr) human papillomavirus (HPV) [49]. The prevalence of HPV is age-dependent since women are infected after cohabitarche and for the majority clearance of the infection is achieved after some years [26]. There are risk factors for HPV acquisition such as sexual intercourse at an early age and multiple sexual partners [43]. Risk factors for high-risk (hr) HPV acquisition are sexual intercourse, young age at sexual debut, multiple partners, oral contraceptive use and smoking [9]. Changes in risk factors and especially the availability of screening can drastically change the incidence of cervical cancer. Screening by cytology has led to a drastic reduction in cervical cancer incidence in high-income countries [41]. This method is not feasible in low-resource settings due to a lack of health service structures, gynecologists and pathology services. It has been shown that screening by visual inspection with acetic acid can also reduce cervical cancer incidence and mortality [29] and is feasible in low-resource settings. Also, vaccination is available to reduce the population-based prevalence of HPV infection by most carcinogenic genotypes and consequently low-grade and high-grade cervical abnormalities [14]. Two recent systematic reviews on cervical cancer screening uptake in Ethiopia found 5% and 13% of women accessing screening, respectively [2, 20]. An important determinant factor for uptake was information provided by health service staff [15, 27]. In Ethiopia, HPV vaccination was introduced in 2018 for school girls in an organised program, and first studies showed an uptake of 80% [1].

### HPV prevalence

More than 206 genotypes of HPV have been discovered of which 14 are considered hr and 6 possible-hr (phr) oncogenic types [8, 44]. A meta-analysis including more than 1 million women with normal cytological findings revealed a global HPV average prevalence of 11.7% for women of all ages. Higher proportions were found in sub-Saharan Africa (adjusted rate of 24.0% for ages 15 and above) [5].

### Young population in rural areas with poor economy and low HIV prevalence

Ethiopia is a country with 115 million habitants and an annual population growth rate of 2.6%. More than half the population is below the age of 15. In Ethiopia, 78% of women (15–49 years) live in rural areas and 48% of all women do not have formal education. The total fertility rate is 4.6 for women aged 15–49; for rural areas, it is 5.2 compared to urban 2.3 [6]. The HIV prevalence is 1.9% in women aged 15-49 years, with 3.7% in urban and 0.6% in rural areas [7]. The economy is fast-growing by annually 10.2% whereas the country’s per capita income in 2019 is still $740 [39]. The population-based registry in Addis Ababa estimates 7,095 cervical cancer patients annually in 2015 [13].

### Cancer control plan and vaccination initiatives at pilot sites in Ethiopia

The Ethiopian Federal Ministry of Health has presented a cancer control plan for 2016–2020. As cervical cancer is one of the top cancers, prevention through vaccination and screening are the main activities [12]. In Ethiopia, there have already been efforts to start cervical cancer screening programs for HIV patients and since 2015 for all women above 30 years. Studies showed that only 10% of HIV-positive women had received screening and only 17% were informed by health workers about screening [37]. Between 2010 and 2014, a number of about 16,000 HIV-positive women were counselled and 99% of those were screened. The service was highly accepted and set the ground for upcoming nationwide activities [35, 36, 48]. Ethiopia introduced visual inspection with acetic acid in the National Cancer Control Plan in 2015. Uptake in a population-based study amounted to 50%, whereas innovative HPV-based self-sampling achieved up to 84% [16, 17].

### Prevalence and types of HPV studied in pregnant women before vaccination initiative started

Vaccination is one of the most cost-effective strategies to prevent HPV-driven cancer [19]. This study aimed to provide information on HPV prevalence in young Ethiopian women attending antenatal care in 2015 before screening was initiated in the country. For logistic reasons and easy collection of vaginal samples, we attempted to include healthy women who visited health centers (avoiding the problems of long-distance travel, lack of privacy, problems with hygiene and so on, in a village setting). Therefore, healthy women attending antenatal care/reproductive services at the health center level were chosen as the study population. We anticipated a younger age structure, more primigravida and a lack of women who do not access formal health services compared to all pregnant women.

## Methods

### Study population

This cross-sectional study on HPV prevalence in vaginal lavage from non-vaccinated, healthy, pregnant women was conducted in 2013 and 2014 in seven centers across Ethiopia.

**Figure 1.**
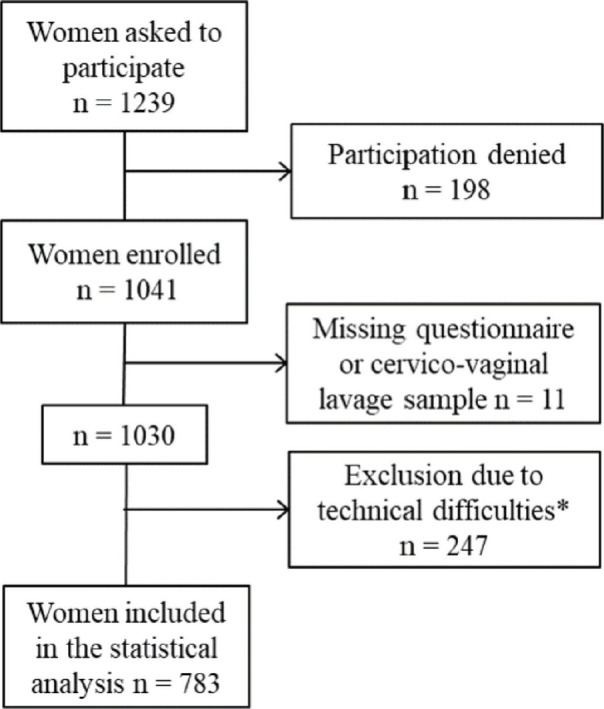
Inclusion of women into the study; * inadequate sample storage *n* = 245; inadequate DNA quality *n* = 2.

To obtain comprehensive data, sites were chosen conveniently from diverse regions (according to common ethnic groups, major religions and urban and rural background). Institutions ranged from university hospitals (*n* = 1), state and private hospitals (*n* = 5) to regional health centers (*n* = 1). Pregnant women from antenatal care (few women attending with unknown pregnancy status) (*n* = 34) were included. Initially, 1,239 women were invited to participate of which 1,041 were enrolled in the study with 783 valid results (Figure 1).

The main inclusion criteria were age 18–45 years, provision of cervicovaginal lavage self-sample and questionnaire. The main exclusion criteria were vaginal symptoms and obstetric conditions such as placenta previa, preeclampsia or recurrent miscarriages. Due to technical problems and operational difficulties, 247 samples were invalid for analysis. A total of 783 women were included in the final population: Addis Ababa *n* = 209, Mekelle *n* = 78, Bahir Dar *n* = 215, Harar *n* = 29, Aira *n* = 149, Wukro *n* = 65 and Ginir *n* = 38. Women who had never lived in an urban setting were considered ‘rural’ assuming that having ever lived in a rural setting could increase the risk of acquiring a persistent HPV infection.

### Questionnaire

The questionnaire used was a modified version that originated from the International Agency for Research on Cancer [11]. Minor adaptations were made to suit the Ethiopian and pregnant populations. It included questions on socio-demographic background, reproductive issues, sexual behaviour, contraceptive methods and medical history. The questionnaire was translated and re-translated from the original English version into Amharic, Oromo and Tigrinya local languages by native speakers of both languages. A brief pre-testing of the questionnaire was done at the first site by ten women who confirmed understanding of the questions (Appendix 1 Questionnaire). The questionnaire was administered and explained through local nurses who were well-known to the clients.

### Sample collection

Samples were collected from April to July 2013 and April to September 2014 during routine antenatal care or family planning clinics. Local nurses obtained informed consent, applied the questionnaire and helped to take the lavage samples. The cervicovaginal lavage sample was collected with assistance through a second-generation self-sampling device according to manufacturer´s instructions (Delphi Bioscience, Scherpenzeel, Netherlands) [10]. Cervicovaginal cells in the lavage obtained with buffered saline were immediately transferred into 3 mL buffered methanol. The samples were stored at room temperature (20°C–30°C) for a maximum of 3 months until transferred to Germany. The samples were stored in Germany at −20°C for a maximum of 6 months.

### HPV DNA testing

HPV DNA testing was performed at the German Cancer Research Center (DKFZ) in Heidelberg, Germany. DNA was extracted from 500 µL lavage fluid using the MagNA Pure 96 DNA and Viral Na Large Volume Kit according to the manufacturer’s recommendations (Roche Diagnostics, Switzerland). To amplify 51 HPV types, a multiplex polymerase chain reaction (PCR) with broad spectrum GP5+/6+ primers was performed followed by bead-based multiplex-papillomavirus-genotyping using the Luminex-reader (Luminex Corp., Austin, TX) as previously described [32]. The detected 51 HPV types included 14 hr- (HPV 16, 18, 31, 33, 35, 39, 45, 51, 52, 56, 58, 59, 66 and 68b), six phr- (HPV 26, 53, 67, 70, 73 and 82) and 31 low-risk (lr) genotypes (HPV 6, 7, 11, 13, 30, 32, 34, 40, 42, 43, 44, 54, 61, 62, 69, 71, 72, 74, 81, 83, 84, 85, 86, 87, 89, 90, 91, 97, 102, 106 and 114). Additionally, three subtypes were integrated (HPV 55, 64 and 68a). Results were expressed as median fluorescence intensities (MFI) of >100 beads per HPV genotype-specific bead class per sample. The cutoff value (5 net MFI) to define DNA positivity was applied as described previously [32]. Determination of β-globin confirmed the presence of DNA. Samples were considered valid if they were positive for β-globin and/or HPV DNA. Viral loads were assessed for all HPV types [24, 30]. Quantification of HPV signals was accomplished by computing the relative HPV MFI signal (%) for each positive reaction by dividing the measured HPV MFI value with the maximum value detected for this HPV type using colony PCR products. Finally, the relative MFI value (%) was divided by the measured β-globin MFI value to form a non-descriptive viral load value (% HPV MFI / β-globin MFI). The high viral load was assessed by a pre-defined HPV type-independent high viral load cut-off of 0.0007 units as described [31].

### Sample size

By attempting to detect a minimum 10% difference between the proportion of HPV-positive women in rural (assumed 14% prevalence and 20% of participants) and urban (assumed 24% prevalence and 80% of participants) areas, a total of 845 women had to be analysed (845 total = 169 rural + 676 urban) alpha 5%, power 80%, *χ*^2^ test. The ratio of rural and urban was derived from a short inquiry into the sites.

### Statistical analysis

SPSS software version 23 (SPSS, Inc., an IBM Company) was used for statistical analysis. Odds ratios with corresponding 95%-confidence intervals (CIs) and *p*-values were calculated by logistic regression analysis for risk factors associated with HPV infection.

### Ethical clearance

Ethical clearance was given by Addis Ababa University in February 2011 (124/10/IM;032/2011), April 2013 (050/2013) and at Martin-Luther-University Halle-Wittenberg, Germany 23.8.2010.

## Results

### Study population

There was a high acceptance among screened women for using the self-sampled cervico-vaginal lavage device (with some assistance by the nurse because of the large abdominal circumference) at the health center during antenatal care visits. During sample collection, 258 women were asked about their experience (samples from the first 3 sites Aira, Wukro and Ginir); 74% mentioned no problem, 20% mentioned discomfort and 6% said it was a bit painful. Key characteristics of the women included with valid samples (783) are presented in Table 1 stratified for whether ever lived in an urban area or always lived in a rural surrounding. Among the 1,025 samples included in this study, 242 (23.6%) had invalid results due to inadequate sample storage.

The age ranged from 18 to 45 years with a mean of 25.9. Most women were reported to be housewives (60%); 15.2% and 13.0% were government employees and merchants, respectively. All Ethiopian major religions and ethnic groups were present in the study population. Educational levels showed only 15.4% and 34.0% illiterate women from urban and rural backgrounds, respectively. The vast majority of women were pregnant (95.7%) of which 10.5%, 35.5% and 54.0% were in first, second and third trimesters, respectively. Almost all women reported to be married (94.9%); nearly half came during their first pregnancy (47.4%). The prevalence of ethnic groups and religions as well as female genital mutilation and polygamy varied between the rural and urban populations. However, age at menarche and sexual debut, parity and marital status were not considerably different in the two groups. Only 31 out of 783 women were ever screened at least once (PAP smear).

### Overall HPV DNA prevalence

**Figure 2.**
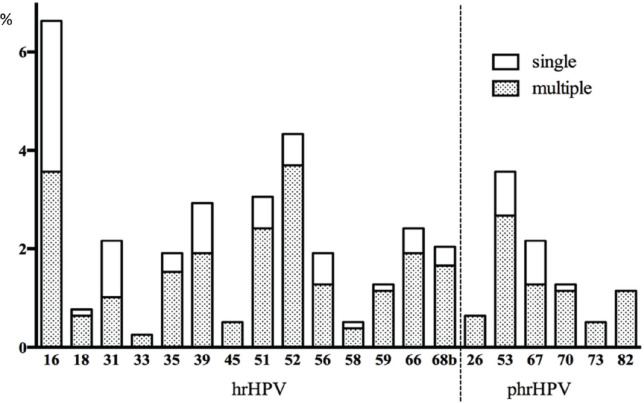
Overview of hr- and phr-HPV infection prevalence (%, *y*-axis) and presence of single or multiple infections in the total population (*x*-axis). Only the white part of the bar chart presents single infections, and the additional shaded part presents multiple infections.

Nearly all samples were positive for β-globin and/or HPV DNA regardless of the amount of fluid collected in the lavage except for 2. The overall prevalence for any HPV genotype was 33.1% (95%CI 29.8–36.4). The prevalence of HPV was considerably different in the rural and urban population with 17.6% (95%CI 11.6–23.7) and 36.8% (95%CI 33.1–40.6) (*p* < 0.001 chi-square test), respectively. Of all, a total of 22.0% were infected with a hr-HPV type of which 62.2% showed a high viral load in at least one hr-HPV infection (13.7% of the total population). Phr- and lr-HPV infections were observed in 7.8% and 18.5% of the women, respectively. Among the targeted HPV types (51) by the assay, 14 hr- and all 6 phr-HPV as well as twenty-two of 31 lr-HPV types were found in one or more samples. Single infections with only one genotype were found in 17.4% (95%CI 14.7–20.0) of women while multiple infections were observed in 15.7% (95%CI 13.2–18.3). In 62 women, simultaneous presence of two HPV types was identified, in 19 women three types, in 23 four types, in twelve five types and in seven women six or more types were found as shown in Figure 2.

HPV prevalence did not differ substantially between pregnancy trimesters. In the first and second trimesters 35.1% and 36.7% of women were positive for any HPV genotype, in the third trimester HPV was a little less frequent (29.7%). The number of women who had been sexually active within the last 3 days was decreasing with increasing trimesters: only 12.7% of women in the third trimester compared to 28.6% and 24.7% in the first and second trimester.

Multiple infections were more common among women with lr- or phrHPV infections (65 of all lrHPV and 75% of all phrHPV positives) than women with hr-HPV infections (59% of all hr-HPV positives). Half of the HPV 16 and 31 infections presented as single infections. The prevalence of HPV types ranged from 6.6% (HPV 16) to 0.1% (HPV 69). The most commonly detected hr-HPV types were HPV 16 (6.6%), 52 (4.3%), 51 and 39 (both 2.9%). HPV 53 as a phr-HPV type was also common (3.6%). Only a few women were positive for the highly oncogenic HPV types 18 and 45 (0.8% and 0.5%). Common lr-HPV types were 81 (2.9%), 62 (2.4%), 42 (2.0%) and HPV 6 (2.0%) (Figure 2).

**Figure 3.**
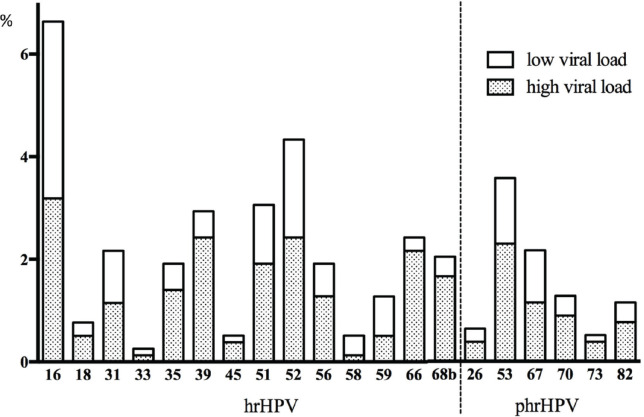
Overview of hr- and phr-HPV infection prevalence (*x*-axis) with low and high viral load in the total population (%, *y*-axis). The high viral load was assessed by a pre-defined HPV type-independent high viral load cut-off of 0.0007 units as described [31]. Only the white part of the bar chart presents a low viral load, the additional shaded part presents a high viral load.

More than half of the patients presented with a high viral load of hr-HPV and phr-HPV (Figure 3).

### Rural and urban HPV DNA prevalence

Of the 15 hr-HPV-positive (single or multiple) women with rural backgrounds 12 had a high viral load (80.0%) while only 95 of the 157 hr-HPV-positive women with urban backgrounds showed high viral loads (60.5%). The distribution of single versus multiple infections did not differ much between the different populations (Table 2).

### Risk factor analysis

Risk factors were chosen according to previous studies: age, educational level, age at first sexual intercourse, use of contraceptives, parity, female genital mutilation and polygamy [43] and origin (rural versus urban) [23]. An exploratory analysis was done for women with urban backgrounds. Smoking is not common among Ethiopian women (*n* = 3) and was not included (Table 3).

The multivariable analysis indicated that women who ever lived in urban areas had a higher risk of being HPV positive with an OR of 2.36 (95%CI 1.47–3.79; *p* < 0.001) (Table 3). Young age at sexual debut (≤15 years of age) also increased the risk of being HPV positive (OR = 1.86 (95%CI 1.02–3.41; *p* = 0.043). This correlation was stronger after stratifying for urban background (OR = 2.18 (95%CI 1.14–4.17; *p* = 0.019). Women reporting that their husbands had more than one wife (polygamy) showed a moderate association with HPV positivity that became apparent when stratified by origin (OR = 1.62 (95%CI 1.00–2.63; *p* = 0.05). Other factors included were not statistically significantly associated with HPV.

## Discussion

In this study, 783 women attending antenatal care in seven sites throughout Ethiopia were included and HPV was detected in one-third of self-sampled cervicovaginal lavages. Participants had a median age of 26 years, one in five had hr-HPV types and one in eight had a high viral load. The most common HPV types were hr-HPV 16, 52 and 51. The prevalence of HPV was 2.3 times higher in women with urban compared to rural backgrounds. Notably, in rural women, the proportion of hr-HPV with high viral load out of all women with hr-HPV was 80% compared to urban women with a proportion of 61%.

A large review summarized publications about women with normal and abnormal cytological findings in Africa (using different detection kits). The prevalence of any HPV type was estimated at 42.2% for East Africa and 12.8% for North Africa [25]. The overall prevalence of HPV in this study is high but not higher than the estimate for East Africa.

However, the prevalence of HPV in this study is higher than other reports from Ethiopia, 15.9% [28], 17.5% [23] and 23.2% [38]. A number of factors account for the difference in prevalence among which the technology used to test HPV and the coverage of the HPV test used are important. The first two studies used the Digene HPV test which tests fewer HPV genotypes compared to ours that targets about 51 different HPV genotypes whereas Teka *et al* [38] used almost similar technology. Here, the screened population was different including older age groups. Our finding is consistent with a report from Ghana [33] that employed a similar technology to test HPV and targeted a similar population of pregnant women. Another Ghanean study showed a prevalence of 32% hr-HPV using the same methodology as this study [22].

In terms of HPV genotypes detected, the top 10 hr-HPV detected from our study participants did not include HPV 18, 58, 33 and 45 reported to be detected from high-grade intraepithelial lesion cases in Africa [25]. Similarly, among the top 10 hr-HPV detected in this study six (HPV16, 35, 52, 31, 56 and 53) were also the top 10 in a population-based study [38]. However, it is always difficult to come into consensus about genotypes circulating in a given area by comparing studies that were made using different techniques among different populations and at different time points.

We compared our results to 3 studies from Ethiopia: the detailed study from rural Attat hospital found HPV type 16 most prevalent followed by 52, 56, 31 and 51 [23]. Wolday *et al* [47] investigated patients at a tertiary referral hospital and found 16, 35, 56, 58 and 18 as the five most common types among cytologically normal women and 16, 45, 31, 35 and 59 among those with cytologic abnormality [47]. Teka *et al* [38] recent population-based study found 16, 35, 52, 31 and 45 as the most common types [38]. HPV 56, 31 and 51 were also among the top 10 prevalent types in our study. In our study, we found 119 of 246 (48,4%) infections covered by the nonavalent vaccine (16, 18, 31, 33, 39, 52 and 58). We conclude that the HPV vaccination program in Ethiopia will effectively reduce the number of women with cervical cancer cases. First pilot sites have been established and school-based vaccination has been proven feasible [45, 46].

We found some individual factors increasing the risk for HPV infection. Women who ever lived in the urban setting had a higher risk of HPV infection with OR 2.36 (1.47–3.79) compared to women who never lived in urban areas. This is similar to the study of Leyh-Bannurah *et al* [23] revealing lower HPV prevalence in women from houses with a traditional roof. In Ethiopia, the urban setting is probably a surrogate marker for a modern lifestyle with more sexual contact of both partners and a known higher HIV prevalence (the number of partners and HIV status were not assessed in our study). A study analysed HIV prevalence in Ethiopia (2005 and 2011 Ethiopian Demographic and Health Surveys). The main risk factors were pre-marital sex, number of life-time sexual partners and high-risk sexual behaviour. These varied considerably between the regions and between ethnic groups explaining regional variation [21]. We postulate that similarly to sexual behaviour and HIV, also HPV prevalence may vary between regions. Our sample size allowed comparison between rural and urban areas but not between regions.

High viral load has been found associated with high-grade cervical intraepithelial neoplasia [42]. In our study, the proportion of women with any HPV type high viral load was 21.2% and women with hr-HPV types high viral load was 13.7%. These women definitely need a follow-up and probably treatment that is usually done only after delivery. Interestingly, in rural areas, the proportion of women with hr-HPV infection with high viral load (12/15) was higher than in women of urban areas (95/157). This could indicate that the rural population had a higher number of high-grade intraepithelial neoplasia and consequently a more advanced HPV infection than the urban population. This definitely shows a need for adequate health service and follow-up.

Early age at sexual debut was associated with a higher prevalence of HPV similar to other studies [43]. Notably early marriage is still rather common in Ethiopia with a median age of 17.1 years among women (age 25–49) in 2011 [6]. This puts a considerable number of women at risk.

The strength of this study is showing HPV prevalence in a representative sample of healthy women in the antenatal care setting as opposed to patients in the hospital setting who do have some kind of health complaint. Self-sampling was well accepted, similar to a study from Ghana [3, 22]. Second, a large number of 783 participants were included from different regions of the country including rural and urban areas. This reflects the diversity in the country to a certain extent considering higher proportions of rural habitants in this study compared to the country.

The limitation is some selection bias since our sample had a small proportion of multi-gravid mothers and women with lower socio-economic backgrounds since they less often attend antenatal care. Therefore, our study missed those women who have more risk factors, leading to possible underestimation in this study. Second, our age-range was narrow, therefore age-standardisation for general comparison was not possible. Of note, our study does not include never-pregnant women. Possibly the group of infertile women (with infertility caused by sexually transmitted infections) could be associated with an even higher proportion of positive HPV status.

## Conclusion

Seeing the high prevalence of HPV in rural and urban populations, there is a clear need for suitable screening and treatment programs. Self- or health worker-based sampling during antenatal care seems feasible to determine HPV and could be a possible, well-accepted option to increase screening uptake using this window of opportunity for screening-eligible women between 30 and 50 years [18]. Linking cervical cancer screening to antenatal care may optimize resources within the health system – although logistic challenges have to be considered since positive women need to be further investigated after delivery. Spontaneous regression after delivery is also possible [2]. Primary prevention through vaccination has started in Ethiopia. The most prevalent HPV16 is covered by the vaccine currently available; the nonavalent vaccine will additionally target HPV 52 as the second most common hr-HPV type in our study. As there was an even higher prevalence in women with urban backgrounds, current trends in migration to cities will probably lead to an increase in the problem. Changes over time will be evaluated.

## Conflicts of interest

The authors have no conflict of interest.

## Informed consent

Ethical approval was obtained from Addis Ababa University Medical Faculty Institutional Review Board (Meeting 050/2013; Protocol 124/10/IM). Written informed consent was obtained from each study participant. All study participants consented orally to being included in the study.

## Author contributions

IR, JMAK, AA and EJK designed the research; JMAK, IR, DHoe, AA SU, CT and EJK analysed the data; JMAK, DHoe and EJK were responsible for drafting the manuscript; JMAK, IR, AKP, TW, SFA, AA, MS, TW and DH provided the original samples, laboratory results and information on the respective populations, and advice on the study design, analysis and interpretation of the findings; all authors provided critical interpretation of the results and review of the first draft; all authors read and approved the final manuscript.

## Data availability

Study data is available on reasonable request from the corresponding author.

## Figures and Tables

**Table 1.** Socio-demographic and reproductive characteristics of the rural, urban and total population, Ethiopia, 2013-2014, *n* = 783.

	Rural	Urban	Total population
N^1^	153	630	783
Age *x̄* (range)	25.2 (18–40)	26.1 (18–45)	25.9 (18–45)
Ethnic group *n* (%)			
Amhara	15 (9.8)	262 (41.7)	277 (35.4)
Oromo	107 (69.9)	106 (16.9)	213 (27.2)
Tigre	26 (17.0)	118 (18.8)	144 (18.4)
Gurage	2 (1.3)	98 (15.6)	100 (12.8)
Other	3 (2.0)	45 (7.2)	48 (6.1)
Religion *n* (%)			
Orthodox	53 (34.6)	438 (69.5)	491 (62.7)
Protestant	91 (59.5)	78 (12.4)	169 (21.6)
Muslim	9 (5.9)	111 (17.6)	120 (15.3)
Other	0	3 (0.5)	3 (0.4)
Education *n* (%)			
Illiterate	52 (34.0)	97 (15.4)	149 (19.1)
Read and write only	25 (16.3)	31 (4.9)	56 (7.2)
Formal education	76 (49.7)	501 (79.7)	577 (73.8)
Menarche *x̄* (range)	14.2 (11–19)	14.8 (7–20)	14.7 (7–20)
Age at sexual debut x̄ (range)	18.7 (9–27)	19.2 (5–33)^2^	19.1 (5–33)^2^
Marital status *n* (%)			
Single	2 (1.3)	20 (3.2)	22 (2.8)
Married	147 (96.1)	596 (94.6)	743 (94.9)
Widowed/divorced	4 (2.6)	14 (2.2)	18 (2.3)
Parity *n* (%)			
Nulliparous	57 (37.3)	314 (49.8)	371 (47.4)
Primiparous	46 (30.1)	168 (26.7)	214 (27.3)
Multiparous	50 (32.7)	148 (23.5)	198 (25.3)
Female genital mutilation *n* (%)	108 (71.1)	330 (53.0)	438 (56.5)
Age at circ. *x̄* (range)	13 (0–20)	5.5 (0–19)	7.4 (0–20)
Formal Polygamy *n* (%)	15 (9.8)	90 (14.3)	105 (13.4)

1 N refers to total number of women in this category. It varies due to missing answers in the questionnaire. Maximum were eight missing answers in one category (circ. circumcision)

2 One woman reported about rape at the age of five

**Table 2.** Prevalence of HPV and HPV genotypes in the rural, urban and total population.

	Rural (*n* = 153)			Urban (*n* = 630)			Total population (*n* = 783)		
	Single HPV	Multiple HPV	Total (%)	Single	Multiple	Total (%)	Single	Multiple	Total (%)
HPV negative	-	-	126 (82.4)	-	-	398 (63.2)	-	-	524 (66.9)
HPV positive	16	11	27 (17.6)	120	112	232 (36.8)	136	123	259 (33.1)
High-risk	5	10	15 (9.8)	65	92	157 (24.9)	70	102	172 (21.9)
Probable high-risk	2	5	7 (4.6)	13	41	54 (8.6)	15	46	61 (7.8)
Low-risk	9	7	16 (10.5)	42	87	129 (20.5)	51	94	145 (18.5)
any HPV with high viral load^1^	10	6	16 (10.5)	82	68	150 (23.8)	92	74	166 (21.2)
hrHPV with high viral load^1^	2	10	12 (7.8)	65	30	95 (15.1)	32	75	107 (13.7)
Proportion of hr HPV with high viral load within hrHPV			12/15 (80)			95/157 (60.5)			107/172 (62.2)
High-risk positive									
16	2	3	5 (3.3)	22	25	47 (7.5)	24	28	52 (6.6)
18	1	0	1 (0.7)	0	5	5 (0.8)	1	5	6 (0.8)
31	0	1	1 (0.7)	9	7	16 (2.5)	9	8	17 (2.2)
33	0	0	0	0	2	2 (0.3)	0	2	2 (0.3)
35	0	1	1 (0.7)	3	11	14 (2.2)	3	12	15 (1.9)
39	0	1	1 (0.7)	8	14	22 (3.5)	8	15	23 (2.9)
45	0	0	0	0	4	4 (0.6)	0	4	4 (0.5)
51	0	1	1 (0.7)	4	18	22 (3.5)	4	19	23 (2.9)
52	0	5	5 (3.3)	5	24	29 (4.6)	5	29	34 (4.3)
56	0	1	1 (0.7)	5	9	14 (2.2)	5	10	15 (1.9)
58	0	0	0	1	3	4 (0.6)	1	3	4 (0.5)
59	0	0	0	1	9	10 (1.6)	1	9	10 (1.3)
66	2	0	2 (1.3)	2	15	17 (2.7)	4	15	19 (2.4)
68b	0	2	2 (1.3)	3	11	14 (2.2)	3	13	16 (2.0)
Prob. High-risk									
26	0	1	1 (0.7)	0	4	4 (0.6)	0	5	5 (0.6)
53	1	0	1 (0.7)	6	21	27 (4.3)	7	21	28 (3.6)
67	1	1	2 (1.3)	6	9	15 (2.4)	7	10	17 (2.2)
70	0	1	1 (0.7)	1	8	9 (1.4)	1	9	10 (1.3)
73	0	0	0	0	4	4 (0.6)	0	4	4 (0.5)
82	0	2	2 (1.3)	0	7	7 (1.1)	0	9	9 (1.1)
Low-risk									
6	0	1	1 (0.7)	1	14	15 (2.4)	1	15	16 (2.0)
11	0	1	1 (0.7)	3	1	4 (0.6)	3	2	5 (0.6)
30	0	2	2 (1.3)	1	10	11 (1.7)	1	12	13 (1.7)
32	0	0	0	1	4	5 (0.8)	1	4	5 (0.6)
40	0	0	0	2	7	9 (1.4)	2	7	9 (1.1)
42	0	2	2 (1.3)	2	12	14 (2.2)	2	14	16 (2.0)
43	0	1	1 (0.7)	0	4	4 (0.6)	0	5	5 (0.6)
44	0	0	0	2	13	15 (2.4)	2	13	15 (1.9)
54	1	0	1 (0.7)	1	9	10 (1.6)	2	9	11 (1.4)
61	0	0	0	1	4	5 (0.8)	1	4	5 (0.6)
62	2	2	4 (2.6)	3	12	15 (2.4)	5	14	19 (2.4)
69	0	0	0	0	1	1 (0.2)	0	1	1 (0.1)
74	0	0	0	3	5	8 (1.3)	3	5	8 (1.0)
81	4	2	6 (3.9)	9	8	17 (2.7)	13	10	23 (2.9)
83	0	0	0	0	3	3 (0.5)	0	3	3 (0.4)
84	1	0	1 (0.7)	1	0	1 (0.2)	2	0	2 (0.3)
86	0	0	0	1	4	5 (0.8)	1	4	5 (0.6)
87	0	0	0	3	2	5 (0.8)	3	2	5 (0.6)
89	0	0	0	2	2	4 (0.6)	2	2	4 (0.5)
90	0	0	0	0	9	9 (1.4)	0	9	9 (1.1)
91	0	0	0	3	5	8 (1.3)	3	5	8 (1.0)
114	1	1	2 (1.3)	3	7	10 (1.6)	4	8	12 (1.5)

1 High viral load: The high viral load was assessed by a pre-defined HPV type-independent high viral load cut-off of 0.0007 units as described [31]

**Table 3.** Risk factors for HPV positivity with odds ratio (OR) and *p*-values using multiple logistic regression.

	All women *n* = 752*				Women from urban areas *n* = 601			
	*n* Total	*n* Positive (%)	Odds ratio	*p*-value	*n* Total	*n* Positive (%)	Odds ratio	*p*-value
	752	246			601	220		
Background								
Rural	151	26 (17.2)	1 (-)					
Urban	601	220 (36.6)	**2.36 (1.47–3.79)**	**<0.001**				
Age								
≥ 30	176	60 (34.1)	1 (-)		144	56 (38.9)	1 (-)	
25–29	268	84 (31.3)	0.83 (0.52–1.31)	0.418	212	76 (35.8)	0.79 (0.48–1.29)	0.350
18–24	308	102 (33.1)	0.78 (0.47–1.3)	0.339	245	88 (35.9)	0.69 (0.4–1.18)	0.172
Education								
No formal education	194	52 (26.8)	1 (-)		120	43 (35.8)	1 (-)	
1–8 years.	220	72 (32.7)	1.24 (0.79–1.95)	0.344	181	66 (36.5)	1.21 (0.74–2.0)	0.451
≥ 9 years.	338	122 (36.1)	1.26 (0.82–1.95)	0.292	300	111 (37.0)	1.14 (0.71–1.83)	0.593
Age at sexual debut								
≥ 21	201	57 (28.4)	1 (-)		160	48 (30.0)	1 (-)	
16–20	471	158 (33.5)	1.35 (0.9–2.01)	0.149	376	143 (38)	1.52 (0.98-2.35)	0.06
≤ 15	80	31 (38.8)	**1.86 (1.02–3.41)**	**0.043**	65	29 (44.6)	**2.18 (1.14–4.17)**	**0.019**
Contraceptives								
No	220	72 (32.7)	1 (-)		173	61 (35.3)	1 (-)	
Hormone contraceptives^1^	532	174 (32.7)	1.02 (0.72–1.45)	0.928	428	159 (37.1)	1.1 (0.75–1.61)	0.622
Parity								
Nulli-/Primiparous	561	191 (34.0)	1 (-)		460	171 (37.2)	1 (-)	
Multiparous	191	55 (28.8)	0.69 (0.42–1.12)	0.128	141	49 (34.8)	0.65 (0.39–1.09)	0.103
Female genital mutilation								
Yes	432	123 (28.5)	1 (-)		324	108 (33.3)	1 (-)	
No	320	123 (38.4)	1.33 (0.96–1.84)	0.086	277	112 (40.4)	1.27 (0.89–1.8)	0.190
Polygamy								
No	654	205 (31.3)	1 (-)		517	180 (34.8)	1 (-)	
Yes	98	41 (41.8)	1.44 (0.91–2.27)	0.117	84	40 (47.6)	**1.62 (1.0–2.63)**	**0.05**

1 Hormone contraceptives include pill, injectable and implant methods; All factors were fitted into a model as nominal and ordinal categories and a reference group was chosen indicated by 1(-).

* Due to missing information 31 subjects of the total cohort were excluded leaving 752 subjects for analysis

## Appendix 1. Questionnaire for HPV prevalence survey

### Risk factor questionnaire

1. Study ID number: |__||__||__||__|

2. Date of interview: *yyyy-mm-dd* - |__||__| -|__||__|

3. Name of interviewer: …………………………………………………………… |__|

4. Type of work |__|

### Demographic data

5. Age (years): |__| |__| *(Woman should be of age 21-40 to participate in study)*

6. Birthplace? Town………………………………………………………………………..|__|

7. Resident now |__||__||__||__|

8. Marital Status: *Single=0, Married=1, Widowed=2, Separated/Divorced=3* |__|

9. How many times have you been married? *(never married=0)* |__|

10. How many years of schooling you have completed? |__|

### Reproductive History

11. At what age did you have your first menstrual period? |__||__|

12. Are you pregnant now? *Yes=1, No=0* |__|

13. How many full-term births did you have before? |__||__|

14. Delivery place: *1=Province clinic, 2=Maternal house, 3=Public hospital, 4=at home* |__|

15. Did you ever have a spontaneous abortion? *Yes=1, No=0* |__|

16. If yes, how many |__||__|

17. Did you ever have a voluntary abortion?*Yes=1, No=0* |__|

18. If yes, how many |__||__|

19. How old is your youngest child? |__||__|

20. How old is your oldest child? |__||__|

### Contraceptive History

21. What method(s) of contraception have you or your partner ever used? Please check all that apply

None |_0_| Birth control Pill |_4_|

Calendar |_1_| Injectable contraceptive |_5_|

Condom |_2_| Tubal ligation |_6_|

Intrauterine device |_3_| Other |_7_|, specify: ………………………………..

*For users of the birth control pill or injectable contraceptive only:*

22. At what age did you start using the contraceptive? |__||__|

23. At what age did you stop using the birth control pill or injectable contraceptive? |__||__| (current age for current users)

24. Are you currently using the contraceptive? *Yes=1, No=0* |__|

### Medical History

25. If women pregnant, how many months since your last period? |__||__|

26. As far as you know, have you ever had a PAP smear? *Yes=1, No=0* |__|

27. Do you have a previous history of cervical cancer or precancer (dysplasia)? *Yes=1, No=0* |__|

28. Have you ever been diagnosed with Tuberculosis? *Yes=1, No=0* |__|

### Smoking History

29. Have you ever smoked regularly? *Yes, currently=1, Yes, but only in the past=2, No, never=0*

|__|

If Yes 30. At what age did you start smoking |__||__|

31. How many cigarettes did/do you smoke per day |__||__|

### Sexual History

32. How old were you when you first had sexual intercourse with a man? Age |__||__|

33. Throughout your life, how many men have you had sexual intercourse with? |__||__|

To your knowledge, did your husband ever have sexual intercourse with another woman 34. before becoming your partner/husband? *1=Yes, 0=No, 2=Don’t know* |__|

35. while being your partner/husband? *1=Yes, 0=No, 2=Don’t know* |__|

36. Does your husband have another wife? How many? |__|

### Blood sampling

37. Blood sample taken: *Yes=1, No=0* specify reason: …………………… |__|

### Gynecological examination

38. Sample taken with self sampler : *Yes=1, No=0* specify reason: …………………………………………|__|

**Number of self-sample specimen …………………………………………………………|__|**
